# *In vitro* Synergistic Activity of Antimicrobial Combinations Against *bla*_KPC_ and *bla*_NDM_-Producing *Enterobacterales* With *bla*_IMP_ or mcr Genes

**DOI:** 10.3389/fmicb.2020.533209

**Published:** 2020-10-21

**Authors:** Chaoe Zhou, Qi Wang, Longyang Jin, Ruobing Wang, Yuyao Yin, Shijun Sun, Jiangang Zhang, Hui Wang

**Affiliations:** Department of Clinical Laboratory, Peking University People’s Hospital, Beijing, China

**Keywords:** Carbapenemase-producing *Enterobacterales*, *in vitro* synergistic activity, triple antimicrobial combinations, double antimicrobial combinations, highly resistant isolates

## Abstract

Carbapenemase-producing *Enterobacterales* have become a severe public health concern because of their rapidly transmissible resistance elements and limited treatment options. The most effective antimicrobial combinations against carbapenemase-producing *Enterobacterales* are currently unclear. Here, we aimed to assess the therapeutic effects of seven antimicrobial combinations (colistin-meropenem, colistin-tigecycline, colistin-rifampicin, colistin-erythromycin, meropenem-tigecycline, meropenem-rifampicin, and meropenem-tigecycline-colistin) against twenty-four carbapenem-producing *Enterobacterales* (producing *bla*_KPC_, *bla*_NDM_, coexisting *bla*_NDM_ and *bla*_IMP_, and coexisting *mcr-*1/8/9 and *bla*_NDM_ genes) and one carbapenem-susceptible *Enterobacterales* using the checkerboard assay, time-kill curves, and scanning electron microscopy. None of the combinations were antagonistic. The combination of colistin-rifampicin showed the highest synergistic effect of 76% (19/25), followed by colistin-erythromycin at 60% (15/25), meropenem-rifampicin at 24% (6/25), colistin-meropenem at 20% (5/25), colistin-tigecycline at 20% (5/25), and meropenem-tigecycline at 4% (1/25). The triple antimicrobial combinations of meropenem-tigecycline-colistin had a synergistic effect of 100%. Most double antimicrobial combinations were ineffective on isolates with coexisting *bla*_NDM_ and *bla*_IMP_ genes. Meropenem with tigecycline showed no synergistic effect on isolates that produced different carbapenemase genes and were highly resistant to meropenem (92% meropenem MIC ≥ 16 mg/mL). Colistin-tigecycline showed no synergistic effect on *Escherichia coli* producing *bla*_NDM__–__1_ and *Serratia marcescens*. Time-kill curves showed that antimicrobial combinations achieved an eradication effect (≥ 3 log_10_ decreases in colony counts) within 24 h without regrowth, based on 1 × MIC of each drug. The synergistic mechanism of colistin-rifampicin may involve the colistin-mediated disruption of bacterial membranes, leading to severe alterations in their permeability, then causes more rifampicin to enter the cell and induces cell death. In conclusion, the antimicrobial combinations evaluated in this study may facilitate the successful treatment of patients infected with carbapenemase-producing pathogens.

## Introduction

During the last decade, carbapenemase-producing *Enterobacterales* (CPE) have gradually become the main pathogen responsible for significant hospital-acquired infections. Because of limited therapeutic options, infections caused by these “super bacteria” are associated with high mortality rates (Logan and Weinstein, 2017). In 2017, the World Health Organization’s priority pathogens list indicated that development of novel antibiotics to treat carbapenem-resistant *Enterobacterales* (CRE) was urgently required (Shrivastava et al., 2018). However, in the current post-antibiotic era, novel antibiotics for treating CRE infections are unavailable owing to the lengthy process of drug discovery and low success rate, which has become a serious concern over the past decade (Luepke et al., 2017). Drug-resistant pathogens are resistant to the most frequently used antibiotics and second-line drugs, resulting in an increased burden of infectious diseases (WHO Pathogens Priority List Working Group, 2018). Thus, antimicrobial combinations may offer an alternative for treating CRE pathogens that are resistant to most available therapies.

Among the many mechanisms that mediate CRE resistance, carbapenemase production is the most common (Nordmann et al., 2011). A series of carbapenemases have been identified in *Enterobacterales*. Three classes of β-lactamases often exist in carbapenem-resistant *Enterobacterales*: Ambler classes A, B, and D. Class A and D β-lactamases have serine-based hydrolytic activities, and class B consists of metallo-β-lactamases with zinc in their active site (Merie Queenan and Bush, 2007). The β-lactamase inhibitors currently available for clinical use consist only of serine inhibitors. For instance, ceftazidime-avibactam works against CRE strains producing *Klebsiella pneumoniae* carbapenemases (KPCs) and OXA-48 but shows no activity against class B β-lactamases. So far, there are no alternative drugs to combat the production of metallo-β-lactamase isolates (Shields et al., 2018).

Combination therapy is a method wherein two or more active antibiotics are used together. This method reduces the frequency of drug resistance and minimizes the dosage of toxic drugs, achieving more significant effects than monotherapy in biochemical activity (Fitzgerald et al., 2006). The Chinese XDR Consensus Working Group (Guan et al., 2016) and most retrospective studies (Nabarro and Veeraraghavan, 2015; Bassetti et al., 2016; Trecarichi and Tumbarello, 2017; Wang et al., 2019) have reported that combination therapy is more effective than monotherapy. However, the advantages of combination therapy remain debatable because different infectious pathogens produce different carbapenemase genes and have different levels of resistance. Investigations focusing on antibiotic combinations that are most effective for treating infections caused by these isolates are limited.

In this study, we explored the synergistic effect of seven antimicrobial combinations against 24 CPE (producing *bla*_KPC_, *bla*_NDM_, both *bla*_NDM_ and *bla*_IMP_, and both *mcr-*1/8/9 and *bla*_NDM_ genes) and one carbapenem-susceptible *Enterobacterales* (CSE) *in vitro*. In addition, the antibacterial synergistic mechanism of colistin with rifampicin was tested using scanning electron microscopy (SEM). The aim of this study was to study the most effective antimicrobial combinations against carbapenemase-producing *Enterobacterales in vitro* activity.

## Materials and Methods

### Microbiological Characteristics of CRE and CSE Isolates

Twenty-five clinical isolates were retrospectively collected from 16 tertiary hospitals in China in 2013–2018 years. The isolates were sent to Peking University People’s Hospital for reappraisal of both resistance mechanisms and antimicrobial susceptibility testing (AST). The isolates were identified by matrix-assisted laser desorption ionization-time of flight mass spectrometry (Bruker Daltonics Inc., Billerica, MA, United States) or a Vitek 2 compact system (BioMérieux Vitek Inc., Hazelwood, MO, United States). Minimum inhibitory concentrations (MICs) were determined by broth microdilution methods according to the CLSI document M100-S30.^1^ For all CPE isolates, polymerase chain reaction (PCR) was used to detect carbapenemase genes (*bla*_KPC_, *bla*_NDM_, and *bla*_IMP_) as previously described (Yigit et al., 2008; Xiaojuan et al., 2014; Khodadadian et al., 2018). The colistin-resistant genes *mcr-*1, *mcr*-8, and *mcr*-9 were also detected by PCR as previously described (Quan et al., 2017; Wang et al., 2018; Yuan et al., 2019). Multilocus sequence typing (MLST) was confirmed according to the Pasteur Institute MLST website^2^ for *K. pneumoniae* and the MLST websites for *Escherichia coli*^3^ and *Enterobacter cloacae*.^4^

### Synergy Testing by Checkerboard Assay

The synergy of double or triple antimicrobial combinations were determined using the standard broth microdilution checkerboard assay as described previously (Berenbaum, 1978; Yoon et al., 2004). In brief, the MICs of antimicrobials were determined before the experiment. Ninety-six-well microtiter plates were arranged with increasing concentrations of one drug, ranging from 0.125 to 8 × MIC on the *x*-axis and increasing concentrations of the other drug ranging from 0.125 to 8 × MIC on the *y*-axis. When using triple antimicrobial combinations, fixed concentrations of the drugs were added into 96-well microtiter plates. The final inoculum in each well was approximately 5 × 10^5^ CFU/mL. The 96-well microtiter plates were incubated at 37°C for 24 h, and turbidity was observed by the naked eye to determine growth. The effects of the antimicrobial combinations were defined according to the fractional inhibitory concentration index (FICI). FICI = (MIC drug A/MIC drug A plus drug B) + (MIC drug B/MIC drug A plus drug B), FICI ≤ 0.5, synergism; 0.5 < FICI ≤ 4, no interaction or FICI > 4, antagonistic (Odds, 2003). With the triple antimicrobial combination, FICI < 1, synergistic, FICI = 1, additive, or FICI > 1, antagonistic (Berenbaum, 1978).

### Static Time-Kill Assay

A static time-kill assay was conducted for four isolates according to the previously described methodology (Lin et al., 2018). Two *Klebsiella pneumoniae* (*bla*_KPC__–__2_, *bla*_NDM__–__1_), 1 *E. coli* (*bla*_NDM__–__1_), and 1 *Serratia marcescens* were selected to examine the bactericidal effects. The double and triple antimicrobial combinations colistin-meropenem, colistin-rifampicin, colistin-tigecycline, colistin-erythromycin, and colistin-meropenem-tigecycline were tested. Bacteria (1 × 10^6^ CFU/mL) were inoculated in Mueller-Hinton broth containing antibiotics with continuous shaking overnight at 35°C and 200 rpm in an atmospheric environment. One hundred microliter samples were drawn and then serially diluted at 0, 4, 8, 16, and 24 h, and 50 μL aliquots were smeared on Mueller-Hinton agar plates. After incubating the plates overnight at 35°C, the colonies were counted. Synergy was defined as a decrease of ≥2 log_10_ CFU/mL between the combination and the most efficient agent alone at 24 h. Bactericidal activity was defined as ≥3 log_10_ CFU/mL reduction in cell numbers compared to the initial inoculum after 24 h (Doern, 2014).

### Scanning Electron Microscopy (SEM)

The colistin-sensitive isolate SF-18-09 was selected to explore the synergistic mechanism of colistin with rifampicin on cellular morphology using SEM as per a previously described method (Zhang et al., 2019). Bacteria at the mid-exponential growth phase (1 × 10^6^ CFU/mL) were added to the final drug concentration according to the checkerboard results, and a no-drug group was used as a control. The cells were incubated for 4 h, as described in the static time-kill assay method. After incubation, samples were transferred to 15 mL polypropylene tubes (Corning, United States) and centrifuged at 10,000 × *g* for 3 min. The supernatants were discarded, and the bacterial pellets were resuspended and washed in 1 mL of 2.5% glutaraldehyde in phosphate-buffered saline (PBS). The tubes were fixed overnight at 4°C. Once fixed, the tubes were centrifuged again at 10,000 × *g* for 3 min, and the supernatants were removed. Bacterial pellets were resuspended in 1 mL PBS and then observed using a scanning electron microscope (Hitachi SU8020).

### Statistical Analysis

Statistical analysis was performed with the software GraphPad Prism version 8.

### Ethical Approval

This study was approved by the research ethics board at Peking University People’s Hospital. As this study was retrospective and participants were anonymized, informed consent was not required.

## Results

### Microbiological Characteristics of CRE Isolates

Genotypic and phenotypic characteristics of CRE and CSE isolates used in this study are displayed in Table 1, including 11 *K. pneumoniae* (6 *bla*_KPC_, 3 *bla*_NDM_, 1 coexisting *mcr*-8 and *bla*_NDM_, and 1 coexisting *bla*_NDM_ and *bla*_IMP_), 6 *E. coli* (4 coexisting *bla*_NDM_ and *mcr*-1, 2 *bla*_NDM_), 5 *E. cloacae* (2 *bla*_NDM_, 1 coexisting *bla*_NDM_ and *mcr*-9, 1 coexisting *bla*_NDM_ and *bla*_IMP_, and 1 non-carbapenemase producer), 2 *K. oxytoca* (both coexisting *bla*_NDM_ and *bla*_IMP_), and 1 *S. marcescens*. The antimicrobials had the following MICs (μg/mL) against all isolates: rifampicin, 8–128; colistin, 0.125–256; meropenem, 0.125–256 (most isolates (23/25) ≥ 16); tigecycline, 0.064–8; and erythromycin, 64–256.

**TABLE 1:** The characteristics of clinical CRE strains in this study.

**Number**	**Bacteria**	**β-lactamase**		**COL-R**	**MLST**	**COL**	**MEM**	**TGC**	**RIF**	**ERY**
		**Class-A**	**Class-B**							
SF-18-03	*kpn*	NDM-9				0.125	64	0.125	128	64
SF-18-04	*kpn*	KPC-2	–	–	ST11	0.25	128	0.25	16	>256
SF-18-09	*kpn*	KPC-2	–	–	ST11	0.25	128	0.25	16	>256
SF-18-33	*kpn*	KPC-2	–	–	ST11	0.25	128	1	16	>256
SF-18-121	*kpn*	KPC-2	–	–	ST11	0.25	16	0.25	16	>256
C3469	*kpn*	KPC-2	–	–	ST11	8	256	4	16	>256
C3497	*kpn*	KPC-2	–	–	ST11	16	256	4	16	>256
SF-18-153	*kpn*	–	NDM-9	–	ST3387	0.25	256	8	32	>256
SF-18-03	*kpn*	–	NDM-9	–	ST520	0.25	64	0.25	128	64
C1376	*eco*	–	NDM-1	–	ST167	2	64	0.125	16	64
C2772	*kpn*	–	NDM-1	–	ST656	8	128	0.5	16	64
C297	*eco*	–	NDM-1	–	ST469	>256	0.125	0.5	128	256
C2550	*ecl*	–	NDM-5	–	ST25	16	16	0.25	16	>256
C3593	*ecl*	–	NDM-5	–	ST1059	2	256	0.25	16	128
C2413	*ecl*		NDM-5 + IMP-4	–	ST256	0.25	128	0.5	16	256
C2896	*kpn*		NDM-5 + IMP-4	–	ST711	0.25	64	8	128	>256
C3012	*kox*	–	NDM-5 + IMP-4	–	–	0.064	64	0.125	128	>256
C2997	*kox*	–	NDM-5 + IMP-4	–	–	0.25	32	0.25	128	>256
C599	*eco*	–	NDM-5	mcr-1	ST10	4	128	1	128	>256
C613	*eco*	–	NDM-5	mcr-1	ST10	4	64	1	128	>256
C1858	*eco*	–	NDM-5	mcr-1	ST10	4	64	0.25	8	>256
C1930	*eco*	–	NDM-5	mcr-1	ST617	4	128	0.125	8	>256
C185	*kpn*	–	NDM-1	mcr-8	ST37	16	32	8	128	>256
SF-18-202	*ecl*	–	NDM-1	mcr-9	ST55	>256	64	0.25	8	>256
SF-18-28	*ecl*	–	–	–	ST365	64	0.032	0.25	16	256
C261	*sma*	–	–	–	–	>256	128	4	32	256

*COL, colistin; COL-R, colistin resistance gene; MLST, Multilocus sequence typing; TGC, tigecycline; MEM, meropenem; RIF, rifampicin; ERY, Erythromycin; MIC, minimum inhibitory concentration; CRE, Carbapenem-resistant Enterobacterales; kpn, Klebsiella pneumonia; ecl, Enterobacter cloacae; eco, Escherichia coli; sma, Serratia marcescens; kox, Klebsiella oxytoca; KPC, K. pneumoniae carbapenemase; NDM, New Delhi metallo-β-lactamase; IMP, imipenemase; mcr, mobile colistin resistance.*

### *In vitro* Evaluation of Synergy Using the Checkerboard Method

We used the broth microdilution checkerboard method to test the following seven antimicrobial combinations: colistin-meropenem, colistin-tigecycline, colistin-rifampicin, colistin-erythromycin, meropenem-tigecycline, meropenem-rifampicin, and colistin-meropenem-tigecycline.

The double antimicrobial combinations of colistin-rifampicin had the highest synergistic effect at 76% (19/25), followed by colistin-erythromycin at 60% (15/25), meropenem-rifampicin at 24% (6/25), colistin-meropenem at 20% (5/25), colistin-tigecycline at 20% (5/25), and meropenem-tigecycline at 4% (1/25). The triple antimicrobial combinations of meropenem-tigecycline-colistin showed a synergistic effect of 100% (16/16). Colistin-tigecycline was ineffective on *E. coli* (Table 2).

**TABLE 2:** Checkerboard results of double and triple antimicrobial combinations for 25 clinical CRE strains.

	**No. of synergy isolates/no. of isolates tested (%)**					
	**By organism**					
**Antimicrobial combination**	***K. pneumoniae* (*n* = 11)**	***E. coli* (*n* = 6)**	***E. cloacae* (*n* = 5)**	***K. oxytoca* (*n* = 2)**	***S. marcescens* (*n* = 1)**	**Total (*n* = 25)**
MEM + TGC	0/11 (0%)	0/6 (0%)	0/5 (0%)	0/2 (0%)	1/1 (100%)	1/25 (4.0%)
MEM + COL	2/11 (18.2%)	0/6 (0%)	2/5 (40.0%)	0/2 (0%)	1/1 (100%)	5/25 (20.0%)
COL + TGC	4/11 (36.4%)	0/6 (0%)	1/5 (20.0%)	0/2 (0%)	0/1 (0%)	5/25 (20.0%)
COL + ERY	7/11 (63.6%)	3/6 (50.0%)	3/5 (60.0%)	1/2 (50.0%)	1/1 (100%)	15/25 (60.0%)
COL + RIF	9/11 (81.8%)	5/6 (83.3%)	4/5 (80.0%)	0/2 (0%)	1/1 (100%)	19/25 (76.0%)
MEM + RIF	2/11 (18.2%)	1/6 (16.7%)	1/5 (20.0%)	2/2 (100%)	0/1 (0%)	6/25 (24.0%)
MEM + COL + TGC	6/6 (100%)	6/6 (100%)	2/2 (100%)	2/2 (100%)	0/0 (100%)	16/16 (100%)

*COL, colistin; TGC, tigecycline; MEM, meropenem; RIF, rifampicin; ERY, erythromycin; CRE, carbapenem-resistant Enterobacterales.*

For CPE isolates, most double antimicrobial combinations were ineffective on the isolates with coexisting *bla*_NDM_ and *bla*_IMP_ genes, including colistin-rifampicin, colistin-meropenem, colistin-tigecycline, and meropenem-tigecycline (Table 3). Meropenem-tigecycline had no synergistic effect on CPE with highly resistant to meropenem. In contrast to colistin-rifampicin, meropenem-rifampicin demonstrated the greatest potential synergistic effect on isolates with coexisting *bla*_NDM_ and *bla*_IMP_ genes, with a synergistic effect of 75% (3/4). Colistin-meropenem also had a synergistic effect of 14.3% (1/7) against *bla*_KPC_-producing isolates and 37.5% (3/8) against *bla*_NDM_-producing isolates. Colistin with tigecycline had no synergistic effect on *bla_NDM__–__1_*-producing *E. coli* and *S. marcescens.*

**TABLE 3:** Checkerboard results of double and triple antimicrobial combinations for 25 clinical CRE strains by CPE.

	**No. of synergy isolates/no. of isolates tested (%)**				
	**By CPE**				
**Antimicrobial combinations**	**KPC (*n* = 7)**	**NDM (*n* = 7)**	**NDM + mcr (*n* = 6)**	**NDM + IMP (*n* = 4)**	**Total (*n* = 24)**
MEM + TGC	0/7 (0%)	0/7 (0%)	0/6 (0%)	0/4 (0%)	0/24 (0%)
MEM + COL	1/7 (14.3%)	2/7 (28.6%)	1/6 (16.7%)	0/4 (0%)	4/24 (16.7%)
COL + TG	2/7 (28.6%)	2/7 (28.6%)	1/6 (16.7%)	0/4 (0%)	5/24 (20.8%)
COL + ERY	5/7 (71.4%)	3/7 (42.9%)	4/6 (66.7%)	2/4 (50.0%)	14/24 (58.3%)
COL + RIF	6/7 (85.7%)	6/7 (85.7%)	6/6 (100%)	0/4 (0%)	18/24 (75.0%)
MEM + RIF	0/7 (0%)	1/7 (14.3%)	2/6 (33.3%)	3/4 (75.0%)	6/24 (25.0%)
MEM + COL + TGC	4/4 (100%)	4/4 (100%)	4/4 (100%)	4/4 (100%)	16/16 (100%)

*COL, colistin; TGC, tigecycline; MEM, meropenem; RIF, rifampicin; ERY, Erythromycin; KPC, K. pneumoniae carbapenemase; NDM, New Delhi metallo-β-lactamase; IMP, integron-encoded metallo-β-lactamase; mcr, mobile colistin resistance; CRE, carbapenem-resistant Enterobacterales; CPE, carbapenemase-producing Enterobacterales.*

### Time-Kill Assay of the Antimicrobial Combinations

**FIGURE 1:**
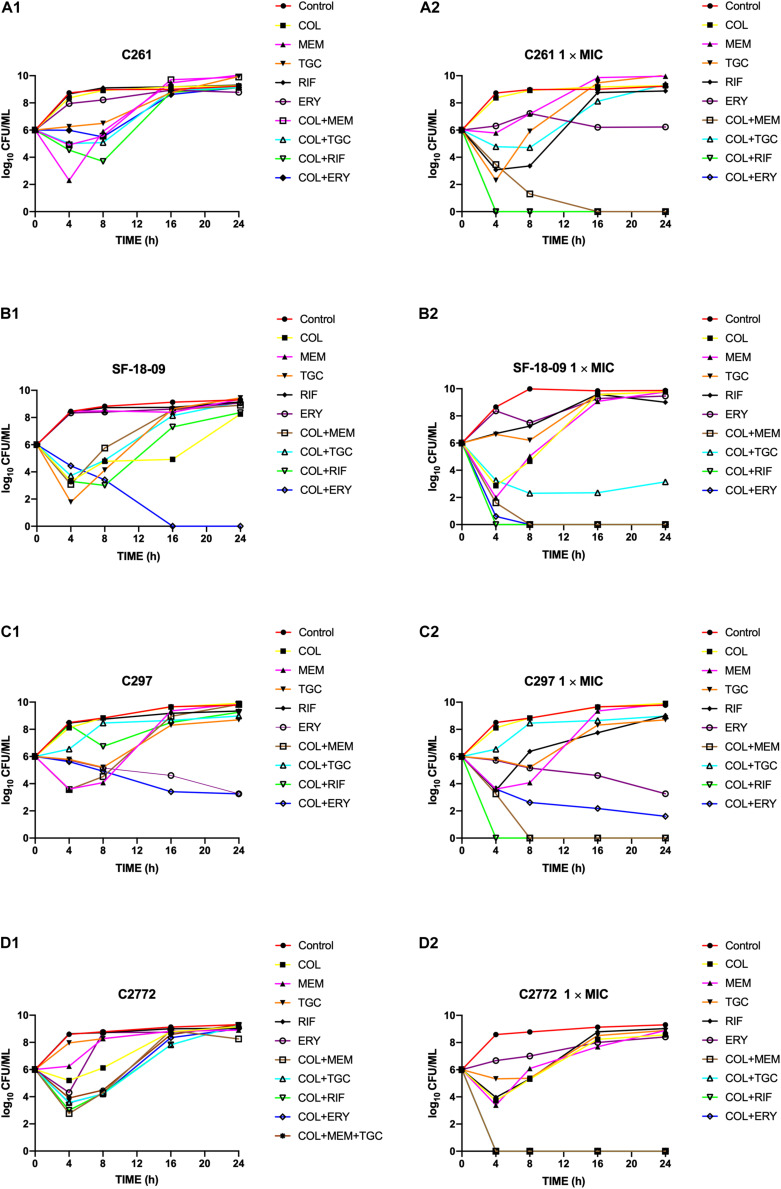
Time-kill curves of colistin alone or in combination with meropenem, tigecycline, rifampicin, and erythromycin against *Serratia marcescens* C261 **(A)**, *Klebsiella pneumoniae* SF-18-09 **(B)**, *Escherichia coli* C297 **(C)**, and *K. pneumoniae* C2772 **(D)**. According to the checkerboard synergistic drug concentration, monotherapy or combination therapy showed no bactericidal effect on clinical carbapenem-resistant *Enterobacterales* (CRE) isolates **(A_1_, B_1_, C_1_, D_1_)**. When using antibiotic concentration 1 × MIC, combination therapy achieved an eradication effect (≥ 3 log_10_ decrease in colony counts) within 24 h without regrowth **(A_2_, B_2_, C_2_, D_2_)**.

Time-kill curves of colistin, tigecycline, meropenem, erythromycin, and rifampicin monotherapy or combination therapy against the two *K. pneumoniae* (SF-18-09, *bla*_KPC__–__2_, C2772, *bla*_NDM__–__1_), one *E. coli* (C297, *bla*_NDM__–__1_), and one *S. marcescens* (C261) are shown in Figure 1. The data represent the changes in bacterial density from an initial inoculum. The antimicrobial combinations that demonstrated synergy via the checkerboard assay were evaluated using the time-kill assay. According to the checkerboard synergistic drug concentration, antimicrobial monotherapy showed no bactericidal effect on all isolates within 24 h. Conversely, the majority of antimicrobial combinations therapies resulted in an early synergistic effect (≥ 2 log_10_ decrease in colony counts) within 4 h. However, the bacteria showed regrowth over 4 h and had the same growth tendency as the control group within 24 h, including those treated with the double (Figures 1A_1_,B_1_,C_1_,D_1_) and triple antimicrobial combinations (Figure 1D_1_). Only the colistin-erythromycin combination showed a bactericidal effect on SF-18-09 within 16 h (Figure 1B_1_).

When using an antimicrobial concentration of 1 × MIC, antimicrobial monotherapy showed no bactericidal effect on all strains within 24 h, whereas the antimicrobial combination therapy achieved an eradication effect (≥ 3 log_10_ decreases in colony counts) over 24 h without regrowth (Figures 1A_2_,B_2_,C_2_,D_2_). Colistin-tigecycline only had a bactericidal effect on C2772 (*K. pneumoniae*, *bla*_NDM__–__1_) isolates (Figure 1D_2_) and a synergistic effect on SF-18-09 (*K. pneumoniae*, *bla*_KPC__–__2_) isolates (Figure 1B_2_), and was ineffective against C297 (*E. coli, bla*_NDM_) (Figure 1C_2_) and C261 (*S. marcescens*) isolates (Figure 1A_2_). In addition, 58.8% (10/17) of the time-kill results were consistent with the checkerboard results.

### Impact of Combination Therapy on Cellular Morphology

As the colistin-rifampicin combination showed the best synergistic effect on CPE isolates, we examined their potential synergistic mechanism. The colistin-sensitive isolate SF-18-09 (*K. pneumoniae*, *bla*_KPC_) was selected for studying the morphological changes in the bacterial cellular surface using SEM. On treatment with a combination of colistin and rifampicin, the cellular surface showed more micelles and deep craters (Supplementary Figure 2D) than in the control group (Supplementary Figure 2A). The cellular surface appeared to burst, causing excessive leakage of the cellular contents. In contrast, colistin monotherapy (Supplementary Figure 2B) caused only slight asperities and craters on the cellular surface. Rifampicin monotherapy (Supplementary Figure 2C) led to the formation of a biofilm layer around the cells, protecting them from being killed.

## Discussion

In this study, we assessed the therapeutic effect of seven antimicrobial combinations (colistin-meropenem, colistin-tigecycline, colistin-rifampicin, colistin-erythromycin, meropenem-tigecycline, meropenem-rifampicin, and colistin-meropenem-tigecycline) against 25 clinical isolates producing different resistance genes (*bla*_KPC_, *bla*_NDM_, coexisting *bla*_NDM_ and *bla*_IMP_, coexisting *mcr-1/8/9* and *bla*_NDM_) and preserving highly resistant to meropenem (92% meropenem MIC ≥ 16 μg/mL) using a checkerboard assay, time-kill curves, and SEM.

Antimicrobial combination therapy aims to achieve bactericidal effects at sub-MICs of the concerned isolates and is important for extending life and reducing economic burden. Colistin is a polypeptide antibiotic that causes rapid bacterial killing in a concentration-dependent manner. It acts on the Gram-negative bacterial cell wall, leading to rapid changes in the permeability of the cell membrane and ultimately cell death (Newton, 1956; Schindler and Osborn, 1979). There are major concerns regarding the safety of colistin doses and the prevention of heteroresistant phenotypes (Owen et al., 2007).

Our current study yielded several notable findings. First, the double antimicrobial colistin-rifampicin combinations showed the highest synergistic effect against all the isolates tested, but was ineffective against isolates with coexisting *bla*_NDM_ and *bla*_IMP_. Although colistin combined with rifampicin is generally regarded as safe for multidrug-resistant *A. baumannii* infections in clinical settings (Bassetti et al., 2008), it is uncertain whether it can be used to treat infections caused by CPE, the *in vivo* evidence remains insufficient.

Colistin-erythromycin had a suboptimal synergistic effect. The antibacterial spectrum of erythromycin mainly targets Gram-positive cocci, with side effects involving liver toxicity and temporary hearing impairment (Mylonas, 2011). Thus, combining colistin with erythromycin may be a feasible method to alleviate its side effects. Although we identified a significant advantage of combining colistin with erythromycin *in vitro*, there is incomplete information in the literature regarding its possible therapeutic effect on CPE infections, and there is a lack of prospective clinical trials to confirm this effect. The potential mechanism of the combination of colistin and erythromycin may be that colistin increases the entry of erythromycin into the cell, playing an indirect role in its bactericidal activity (Vaara, 1992; Ofek et al., 1994).

The combination of meropenem with tigecycline had no synergistic effect on CPE with highly resistant to meropenem. This result is consistent with a recently published study, which reported that combination with meropenem is becoming less effective against strains with meropenem MIC > 8 μg/mL (Del Bono et al., 2017).

Triple antimicrobial combinations are being considered as a treatment option against serious CPE infections, and have shown promising results *in vitro* (Diep et al., 2017). In our study, the triple antimicrobial combinations of meropenem-tigecycline-colistin showed a synergistic effect of 100%. This is the first study demonstrating the effectiveness of triple antimicrobial combinations against coexisting carbapenemase gene isolates, and more clinical trials are required to validate their effectiveness.

We also confirmed the checkerboard results using time-kill assays, which provided dynamic measurements of bactericidal activities over time to explore the *in vitro* bactericidal effects. Four strains were selected for this analysis. According to the checkerboard synergistic drug concentration, unsatisfactory bactericidal activity was observed against all strains within 24 h when isolates were treated using either monotherapy or combination therapy, and the same growth tendency was observed as in the control group. However, these results are in contrast with those of another study conducted by Soudeiha et al. (2017), showing that antimicrobial combinations at sub-MIC levels can also prevent bacterial regrowth. The discrepancy between these results may be explained by the use of isolates with different carbapenemase-producing and resistant levels. When using antimicrobial concentrations of 1× MIC, the combination of antimicrobials achieved an eradication effect (≥ 3 log_10_ decreases in colony counts) by 24 h without regrowth compared with monotherapy, which showed regrowth. The combination of colistin with other antimicrobials also show bactericidal effects on *S. marcescens* strains that are intrinsically non-susceptible to colistin, as several classes of available antibiotics can penetrate the envelope barrier effectively in the presence of colistin (Fajardo et al., 2013). We firstly found that colistin-tigecycline had no synergistic bactericidal effect on *bla*_NDM__–__1_-producing *E. coli* and *S. marcescens.*

SEM was used to observe morphological changes in the bacterial cellular surface. Rifampicin monotherapy resulted in the production of a layer of biofilm formation around cells, protecting them from death. Previous reports have demonstrated that the ability of bacteria to form biofilms may contribute to treatment failure as biofilm-forming bacteria are less susceptible to antibiotics (Donlan and Costerton, 2002). Colistin combined with rifampicin caused more micelles and deep craters than monotherapy, which has been shown to be a precursor of cell death according to the carpet model hypothesis (Ciumac et al., 2019). We also observed structural damage via toroidal pore formation, followed by damage to the bacterial membrane and cell death. This phenomenon strongly supports the notion that the synergistic mechanism of colistin with rifampicin may involve changes in the outer cell membrane permeability encoded by colistin, allowing more rifampicin to enter and kill cells. Another possible mechanism is that the combination of colistin with rifampicin reduces the viability of the cell biofilm at low rifampicin concentrations (Geladari et al., 2019).

In conclusions, we found that the double antimicrobial combinations of colistin with rifampicin had the highest synergistic effect on isolates that produced different carbapenemase genes and were highly resistant to meropenem. However, this combination was ineffective on isolates with coexisting *bla*_NDM_ and *bla*_IMP_ genes. The triple antimicrobial combinations of meropenem, tigecycline, and colistin had a synergistic effect of 100%. Colistin with tigecycline had no synergistic effect on *bla*_NDM__–__1_-producing *E. coli* and *S. marcescens*. The limitations of this study include the lack of *in vivo* experiments conducted, in addition to its limited sample sizes. Whether these *in vitro* findings can be applied to a clinical setting needs to be confirmed in further studies, including PK/PD (pharmacokinetics/pharmacodynamics), *in vivo* experiments, and prospective randomized clinical trials. In general, the antimicrobial combinations evaluated in this study may facilitate the successful treatment of patients infected with Carbapenemase-producing *Enterobacterales*.

## Data Availability Statement

The raw data supporting the conclusions of this article will be made available by the authors, without undue reservation, to any qualified researcher.

## Author Contributions

HW conceived and designed the study and revised the draft. CZ, QW, LJ, RW, YY, JZ, and SS performed the experiments described in this study. CZ performed the statistical analysis and wrote the draft. All authors approved the final version of the manuscript.

## Conflict of Interest

The authors declare that the research was conducted in the absence of any commercial or financial relationships that could be construed as a potential conflict of interest.

## Footnotes

1 http://www.clsi.org

2 http://bigsdb.pasteur.fr/klebsiella/klebsiella.html

3 http://mlst.warwick.ac.uk/mlst/dbs/Ecoli

4 https://pubmlst.org/ecloacae/
