# Supplementary material for: In vitro Synergistic Activity of Antimicrobial Combinations Against blaKPC and blaNDM-Producing Enterobacterales With blaIMP or mcr Genes
Source: Front Microbiol. 2020 Oct 21;11:533209. doi: 10.3389/fmicb.2020.533209 (PMC7609915; doi:10.3389/fmicb.2020.533209)
Supplement: Supplementary file 1 [file Data_Sheet_1.PDF]

**Supplemental materials**

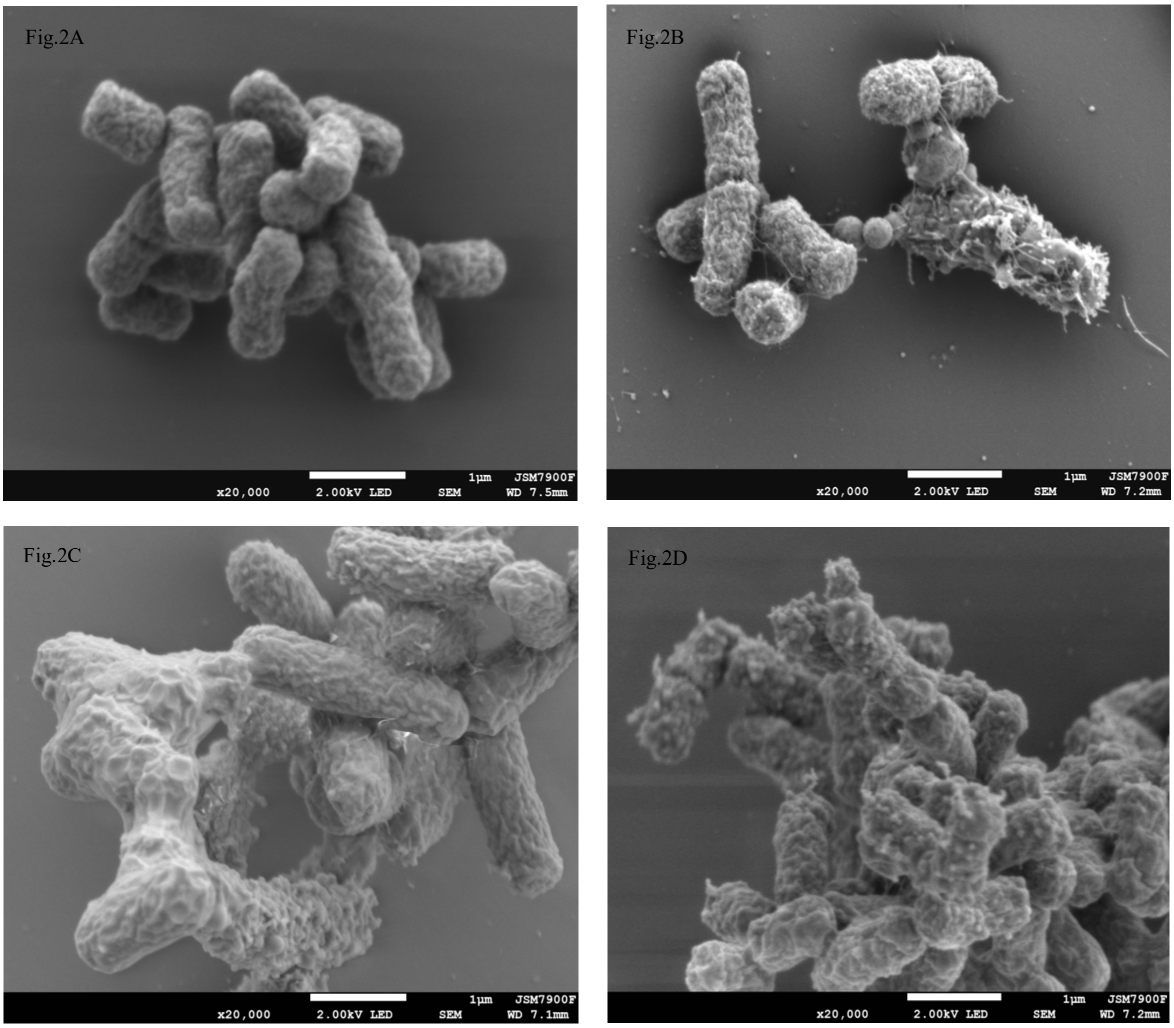


**Figure1.** Scanning electron microscopy (SEM) was used to study the synergistic mechanism of the combination of colistin with rifampicin. the combination therapy revealed swelling of the bacterial cell surface and showed deep craters (Fig.2D) compared with a control group (Fig.2A); monotherapy colistin (Fig.2B) caused slight surface aberrations and a few craters. Monotherapy rifampicin (Fig.2C) showed a layer around the cells protecting them from killing.
